# Artificial intelligence in the prediction of protein–ligand interactions: recent advances and future directions

**DOI:** 10.1093/bib/bbab476

**Published:** 2021-11-27

**Authors:** Ashwin Dhakal, Cole McKay, John J Tanner, Jianlin Cheng

**Affiliations:** Department of Electrical Engineering and Computer Science, University of Missouri, Columbia, MO, 65211, USA; Department of Biochemistry, University of Missouri, Columbia, MO, 65211, USA; Department of Biochemistry, University of Missouri, Columbia, MO, 65211, USA; Department of Chemistry, University of Missouri, Columbia, MO, 65211, USA; Department of Electrical Engineering and Computer Science, University of Missouri, Columbia, MO, 65211, USA

**Keywords:** protein–ligand interaction, drug discovery, binding site, binding affinity, binding pose, machine learning, deep learning

## Abstract

New drug production, from target identification to marketing approval, takes over 12 years and can cost around $2.6 billion. Furthermore, the COVID-19 pandemic has unveiled the urgent need for more powerful computational methods for drug discovery. Here, we review the computational approaches to predicting protein–ligand interactions in the context of drug discovery, focusing on methods using artificial intelligence (AI). We begin with a brief introduction to proteins (targets), ligands (e.g. drugs) and their interactions for nonexperts. Next, we review databases that are commonly used in the domain of protein–ligand interactions. Finally, we survey and analyze the machine learning (ML) approaches implemented to predict protein–ligand binding sites, ligand-binding affinity and binding pose (conformation) including both classical ML algorithms and recent deep learning methods. After exploring the correlation between these three aspects of protein–ligand interaction, it has been proposed that they should be studied in unison. We anticipate that our review will aid exploration and development of more accurate ML-based prediction strategies for studying protein–ligand interactions.

## Introduction to protein–ligand interactions

**Figure 1:**
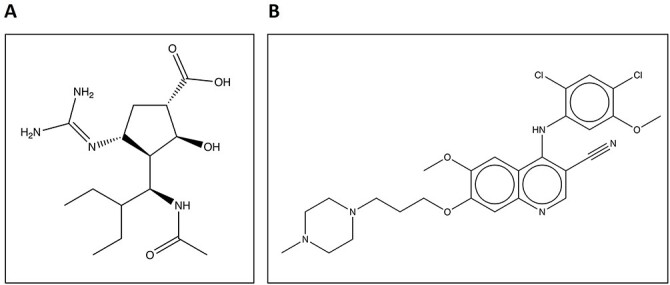
(**A**) Sketch of peramivir, an inhibitor of the viral protein neuraminidase from the H1N9 influenza virus. (**B**) Sketch of human Src kinase inhibitor bosutinib.

Proteins participate in a wide range of essential intra- and intercellular mechanisms. However, they do not work independently in living organisms. Frequently, they must bind with other molecules (other proteins, nucleic acids, metal ions, organic and inorganic molecules, etc.) to form a specific interaction in order to perform their function [1–4]. Species capable of binding to the protein are known as ligands. As an example, consider the inhibitory drugs peramivir and bosutinib, as demonstrated in Figure 1, which will be used to illustrate protein–ligand interactions in the subsequent sections.

Particularly, intermolecular interactions between proteins and ligands occur at specific positions in the protein, known as *ligand-binding sites*, which has sparked a lot of interest in the domain of molecular docking and drug design. Binding sites, also referred to as binding pockets, are typically concavities on the surface of proteins. Pockets, where small drug-like ligands bind, are typically located in deep cavities. Ligand-binding sites are typically found in large, deep pockets [5, 6] on the protein surface, while some of them may exist in exposed shallow clefts [7, 8]. In medicinal chemistry, there is an emphasis on identifying key proteins whose biochemical functions can be definitively linked to diseases. Such proteins become targets for drug development. In fact, the binding site is considered druggable if the ligand binds with high affinity at the binding site and has an effective therapeutic action [9].

**Figure 2:**
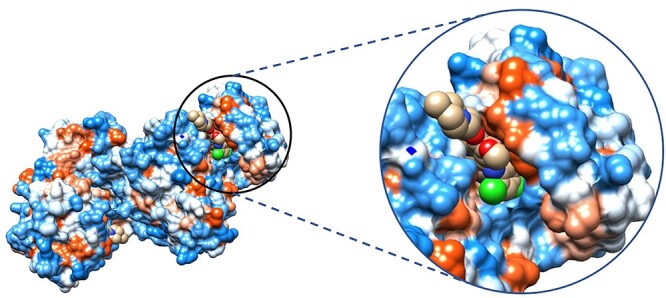
Human Src kinase docked by bosutinib visualized with a hydrophobic surface generated in Chimera, PDB code 4MX0. Most hydrophobic regions colored red; most hydrophilic indicated in blue.

When attempting to predict protein–ligand interactions, a labyrinth of interactions needs to be accounted for to generate an accurate prediction. Biologically, two major factors play into the complexity of protein–ligand interactions, large spectrum of ligand types: small organic molecules, organometallics, nucleic acids, peptides and even other proteins [10]. This paper will primarily focus on small organic compounds as those are immediately relevant to medicinal therapies as this class of ligands is more commonly associated with inhibitor and inactivating ligands than other classes of ligands. The second factor is the resulting intermolecular forces from within the protein–peptide chains, protein-solvent interactions and the binding ligands [10, 11]. Often, these forces are represented by forcefields to simplify computation. However, some estimations resulting from the use of forcefields have been scrutinized as a means of error when generating predictions for ligand interactions and has been shown in the past to significantly misrepresent the potential binding affinity, poses of the suspected ligands [11]. Due to the large variety of ligand types, the defining interactions between protein and ligand can also vary. The most recognizable stabilizing forces are hydrogen bonding and the columbic forces also more commonly referred to as electrostatic interactions. This however is far from the only intermolecular forces driving favorable binding enthalpy. Van der Waals forces including the critical hydrophobic interactions of the London dispersion forces, pi stacking of aromatic compounds and other ion-induced dipole and dipole–dipole interactions also play a role [10, 11]. An example of this can be seen with the human Src kinase inhibited by bosutinib. To better visualize this mode of binding between Src kinase and bosutinib, a hydrophobic surface rendering was generated in chimera as seen in Figure 2. In addition, these images showcase the ligand, bosutinib, drawn in the space fill style to better demonstrate why the ligand is posed in the way that it is as not only do the interactions need to be fulfilled, but they must also satisfy steric requirements to avoid clashes. Another major interaction is the energy involved in overcoming the desolvation of the ligand and the binding residues as they interact with the solvating water molecules. These forces are not static in their accumulative contributions to binding enthalpy. For example, hydrogen bonding can vary greatly ranging from 1 up to 40 kJ/mol due to factors like the donating and accepting species, the type of hydrogen bonding and the distance between the donating and accepting species.

The production of a new drug, from target identification through approval for marketing, can take over 12 years and cost around $2.6 billion [12–14]. The COVID-19 pandemic has unveiled the urgent need for rapid drug development [3]. In most drug design projects, the initial goal is to find ligands that bind to a specific protein target with high affinity and specificity. There is a significant need for expediting the computational process for identifying promising drug candidates for experimental validation [15].

An initial step in the drug discovery pipeline is to identify molecules that bind with high affinity to the target, which can be further developed into drug-like molecules (lead compounds) [16]. Because of our limited understanding of the dynamic relationship between chemical space and genomic space [17–19], identifying novel drugs and their targets remains a difficult task. Experimental methods to identify lead compounds, such as high-throughput screening, can be time-consuming and expensive [16]. In contrast, computational prediction of protein–ligand interaction (PLI) can significantly reduce the resources, time and cost required and reduce the need for physical experimental studies to screen for new therapeutics. Reliable PLI predictive algorithms can thus greatly accelerate the development of new treatments, remove toxic drug candidates and efficiently direct medicinal chemistry [20]. Machine learning (ML) algorithms adopt a different approach from classical virtual screening (VS) [21] approaches. In the case of ligand-based virtual screening (LBVS), it utilizes the active ligand’s information and similarity between candidate ligands and the known active compounds to find new ligands [22]. As a result, these methods are useful when there is no 3-dimensional structure of the target protein available. Likewise, structure-based virtual screening (SBVS) method uses the 3D structure of a target to screen compound libraries [23]. In contrast, ML follows the approach of learning the relation between physicochemical parameters and protein–ligand interactions from the known structures of protein–ligand complex pairs to derive statistical models for predicting the status of other unknown ligands/proteins.

To identify associations between drugs and target proteins (i.e. interaction between them), Yamanishi *et al.* [24] suggested a kernel regression-based technique to infer protein–ligand interactions by combining the chemical structure information of ligands, sequence information of proteins, as well as the drug–target complex network. Similarly, another published work includes the experiments by Bleakley and Yamanishi [25], called BLM, that employs the supervised learning method. Cao *et al.* [26] proposed another prediction method based on the random forest (RF) algorithm. Similarly, in the framework of restricted Boltzmann machines, Wang and Zeng [27] introduced the method to predict not just binary contacts between proteins and ligands but also diverse types of interactions, viz how they interact with one other. Readers can explore the review papers [28–31] for the AI-driven drug discovery process including target identification, hit identification, lead optimization, chemical synthesis prediction and drug repositioning.

**Figure 3:**
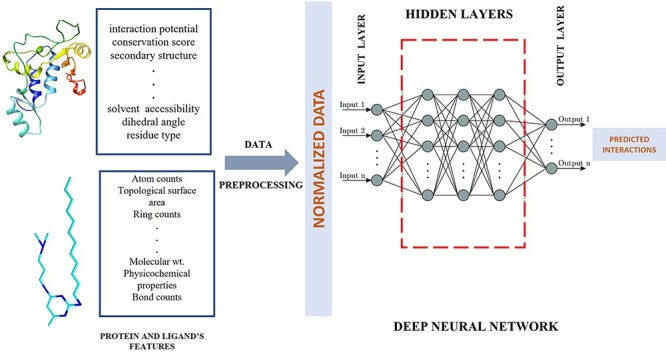
Conceptual workflow of ML pipeline. Inputs are the properties of the target protein and ligands, and output are the predicted interactions.

A simplified illustration of ML technique in protein–ligand interaction prediction is depicted in Figure 3. This figure represents the general workflow of ML architecture. Initially, the features of target protein and ligand are extracted, followed by data preprocessing steps. Normalization (a data preparation technique) is frequently used in machine learning, which converts the values of numeric columns in a dataset to a similar scale without distorting the ranges of values or losing information. Normalized data, thus obtained, are fed to a machine learning model such as neural network in the figure. This step is often performed because data standardization improves accuracy, as shown by empirical evidence [32]. Several processes occur at hidden layers and eventually the output layer outputs the predicted decision. As it does not demand the explicit hard-coded rules curated by human experts to make prediction and potentially yield good prediction accuracy, there has been a lot of interest in using ML and particularly powerful deep learning methods to predict PLIs.

To achieve highly accurate predictions for new data, a sufficient amount of training data is required. The increase of structural information for protein–ligand complexes, and the cataloging of this information into databases, has enabled researchers to explore artificial intelligence (AI) approaches, mostly ML methods, for virtual screening.

Owing to the advent of many robust AI techniques and the abundance of data in this domain, many surveys have been conducted examining the existing ligand-binding site, binding affinity and binding pose prediction algorithms. A decade ago in 2009, a review group led by Henrich, analyzed various computational methods to identify protein-binding sites for ligand design [33]. Because machine learning applications in this field were still in their infancy years ago, authors focused mostly on the traditional, yet successful, methods. Macari *et al.* [34] published a review paper focusing on the computational paradigms in the domain of protein-small molecule binding site prediction. Here, they analyzed the approaches from traditional geometrical techniques to recent machine learning strategies and compared the characteristics and performances of the techniques. Similarly, Zhao and his team [35] discussed the expensive computing resources associated with training deep learning algorithms in comparison to traditional machine learning algorithms with an ending note that the prediction problems are still not solved mainly because of presence of some cryptic sites [36].

Focusing on the binding affinity (inhibition constant, dissociation constant and binding energy) prediction models, the team of Heck (in 2017) published a review paper largely concentrating on successful supervised machine learning methods. Authors mention that this holistic credit behind the rapid development of ML strategies in this field goes to the open-source ML libraries and the publicly available data sources, while Yang’s group (in 2020) [37] argue that sufficiently large and unbiased datasets would help training robust AI models more accurately to predict protein–ligand interactions.

In the paper by Ellingson *et al.* [38], the authors discuss the trends of ML in the domain of drug-binding prediction (binding pose and energy prediction), data sources and potential problems associated with them. Additionally, Chen’s group [39] summarizes the web servers and databases used in drug–target identification and drug discovery. Here, for ML-based approaches, they mostly concentrate on the supervised and semisupervised models. A similar kind of analysis was carried out by Inhester and Rarey [40] describing the publicly available databases containing the affinity data and structural information that plays a vital role in describing interaction geometries and strength of binding.

Recently, Lim’s team [41] published a review paper on compound protein interaction (CPI) prediction models that includes a precise description of the data format used, the techniques associated with model development and emerging methods. They also provide an overview of databases as chemistry-centric, protein-centric and integrated database and analyzed the diversified methods of AI like, tree, neural network, kernel and graph-based methods in the field of CPI. Since widely used human-readable formats, SMILES, generally fail to represent critical information like neighborhood in 3D space, latent vector representation of compounds and proteins is highly recommended. In the same way, since deep learning (DL) methods make black-box decisions (difficult to understand how decisions are made by neural networks), authors are in favor of attention mechanisms to address this issue.

**Figure 4:**
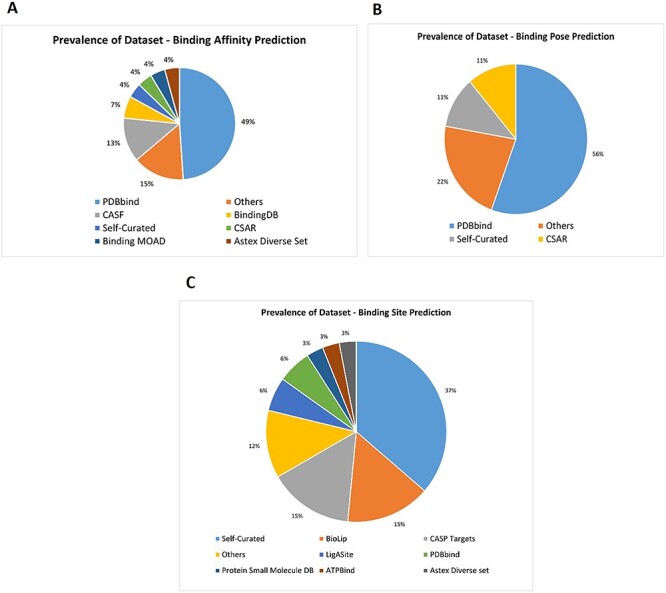
Pie charts showing the distribution of prevailing datasets for the AI-based PLI prediction models. (**A**) Prevalent dataset for AI based protein-ligand binding affinity prediction models. (**B**) Prevalent dataset for AI based protein-ligand binding pose prediction models. (**C**) Prevalent dataset for AI based protein-ligand binding site prediction models.

The unique goal of this review is to illustrate the importance of interconnection of separately discussed topics of PLI: binding site, binding affinity and binding pose prediction beforehand. A systematic search on three aspects of ML-based protein–ligand interactions (i.e. binding site, binding affinity and binding pose prediction) was carried out using Google Scholar. Research methodologies were populated based on the year of publication as well as underlying relevant techniques. Selected literature was analyzed, articles and group of articles were compared, themes identified, and gaps noted, and suggestions recommended for future research. We have tried to provide a comprehensive, organized summary of related databases, recent research trends in AI-guided PLI prediction, their interconnection and prospects so that researchers can fully utilize these resources to develop novel prediction methods.

## Existing databases for AI-driven protein–ligand interaction models

The main purpose of ML/AI algorithms is to reveal hidden information/knowledge in data. For ML models to understand how to perform different tasks, training datasets are fed into the algorithm. The model sees and learns from the training data automatically. The model can recognize the underlying, hidden relationships and patterns in the data that are not obvious to human and even experts. The validation dataset, on the other hand, is a different dataset that is often used during training to assess how well the model is performing and used to tune the hyperparameter of the model. After the model has been fully trained and validated, one can run assessment metrics on an independent test dataset not used in training to monitor the performance of the model predictions. As a result, data are critical for such ML applications. Generally, the more data provided to the ML system, the greater its performance of learning and prediction.

Here, we discuss the prevailing datasets in the field of PLIs that serve as sources of training, testing and validating data for ML/AI methods. We studied 63 AI-driven methods that have been published since 2004 and investigated the availability of several databases for training–testing–validating them. In Figure 4A, 32 publications on binding affinity prediction using AI’s most frequently used database is PDBBind [42], followed by CASF benchmarking dataset [42] and BindingDB [43]. In Figure 4B, we observe the recurrence of PDBBind for training AI models for predicting binding pose that signifies its popularity and usefulness. As shown in Figure 4C, out of 25 AI-driven binding site prediction methods, many datasets are mostly self-curated by developers according to the requirement for training–testing–benchmarking purposes. The most frequent datasets used in this domain are BioLip, CASP targets, LigAsite and PDBbind. It is worth noting that many datasets were created using the original protein structure and ligand data in the Protein Data Bank (PDB) [44]. Overall, these datasets would be valuable resources for those who are looking for validating and developing ML-driven PLI prediction methods and for the study of drug design in general.

### PDBBind

**Figure 5:**
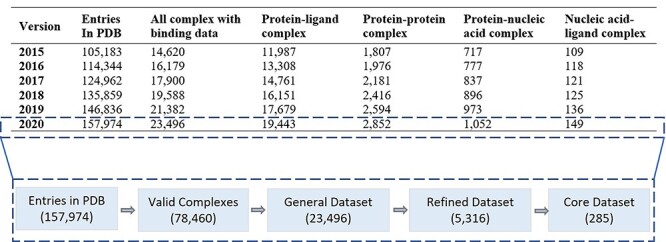
Statistics of PDBBind dataset showing its composition from Version 2015 as well as the basic structure of Version 2020.

The PDBbind database was created in 2004 by Wang *et al*. [42] providing a broad set of binding affinity data that are experimentally determined. The binding affinity data are for all the types of biomolecular complexes that are deposited in the PDB. Originally, PDBbind was limited to complexes formed by proteins and small-molecule ligands. Starting from 2008, other types of biomolecular complexes in PDB were added into PDBbind. Being updated annually, the latest release (i.e. version 2020) contains binding data (K_d_, K_i_ and IC_50_ values) for 19 443 protein–ligand, 2852 protein–protein, 1052 protein–nucleic acid and 149 nucleic acid–ligand complexes as shown in Figure 5. Here, all binding data are curated by the authors derived from original literatures.

The PDBbind version 2020 is based on the contents of PDB officially released at the first week of 2020. It contains 157 974 structures that were experimentally determined. A series of computer programs were implemented to screen the entire PDB to identify four major types of molecular complexes: protein–small ligand, nucleic acid–small ligand, protein–nucleic acid and protein–protein complexes. Version 2020 is the most recent edition at the time of writing this manuscript.

### LIGand attachment site (LigASite)

LIGand Attachment SITE (LigASite) is a publicly accessible dataset of biologically relevant binding sites in protein structures. To automatically filter out the biologically irrelevant ligands, an algorithm is used that considers the number of heavy atoms in the ligand and the number of interatomic contacts between protein and ligand [45]. The fact that each protein has both unbound and bound structures ensures that this dataset can be used to benchmark the binding site prediction models. The version 9.7(nr25) contains the list of 391 proteins, while the redundant list contains the information of 816 proteins.

### BioLiP

BioLiP is a semimanually curated database of ligand–protein binding interactions that are biologically relevant. After the automated process for determining a ligand’s biological relevance is completed, a thorough manual review is performed to correct any errors. The structure data are mainly obtained from the PDB, with biological insights obtained from literature and other databases. Eventually, the manual check is performed, and possible false-positive entries are verified by reading the original literature and consulting other databases that ensures the completeness and high quality of BioLiP [46]. It is updated on weekly basis and the total number of entries in the current version (17 September 2021: at the time of writing) is 529 047, which includes 109 998 proteins from PDB, 57 059 DNA/RNA ligands, 25 960 peptide ligands, 146 969 metal ligands and 299 051 regular ligands. Out of these entries, 23 492 have binding affinity information.

### BindingDB

BindingDB is a public, web-accessible database, extracted from the scientific literature, which consists of binding affinities between protein targets and small, drug-like molecules. The latest version: (September 2021), it has 41 296 entries, containing 2 338 906 binding data with 8617 protein targets and 1 011 134 small molecules [43]. Moreover, the BindingDB website offers a comprehensive collection of tools for querying, analyzing and downloading binding data.

### Binding MOAD

Binding MOAD is the largest possible (hence called mother of all databases) deposition of high-quality, protein–ligand complexes available from the PDB, which was augmented with the inclusion of binding data extracted from literature. Binding MOAD’s preference for affinity data is K_d_ over K_i_ over IC_50_ [47–49]. It was designed using the ‘top-down’ approach so that it contained every protein–ligand complex with a 3D structure. Annual updates are done to contain more binding-affinity data as they become available in the PDB. The current release (2019) contains 38 702 protein–ligand structures, 14 324 binding data, 18 939 ligands and 10 500 protein families.

### The CASF dataset

Scoring functions are often used to evaluate PLIs in structure-based drug design. Several scoring functions have been established thus far, and hence some open-access benchmarks are required for assessing their strength and weakness. CASF benchmark provides the precalculated prediction results of known scoring functions allowing a fair comparison of the model with existing scoring functions on the same test set. All performance tests enabled in CASF-2016 are based on a set of 285 protein–ligand complexes with high-quality crystal structures and reliable binding data [42]. This test set is selected from the PDBbind refined set (version 2016). In CASF-2016, the evaluation methods have been improved in comparison to its previous version (CASF-2013) and the performance of a scoring function is evaluated by four metrics: (i) scoring power, (ii) ranking power, (iii) docking power and (iv) screening power.

## Prediction of the ligand bind sites of proteins

Virtual Screening (VS) requires knowledge of the location of the ligand-binding site (LBS), which in some cases this information is unknown. Accurate prediction of protein–ligand binding sites from a 3D protein structure plays a crucial role in structure-based drug design [50, 51] and can aid in drug side effects prediction [52] as well as understanding a protein’s function [53]. Intermolecular interactions between proteins and ligands occur through amino acid residues at specific positions in the protein, usually located in pocket-like regions. Identification of these key residues is imperative for elucidating protein function, analyzing molecular interactions and facilitating docking computations in virtual screening–based drug design. These specific key amino acid residues in proteins are called the LBSs. Empirical studies show that the actual ligand-binding site correlates to the biggest pocket on the surface of a protein [6, 54]. On a test set of 67 protein structures [55], the SURFNET architecture [56] successfully predicted the ligand-binding site as the largest pocket in 83% of the cases. The findings from LIGSITE [57] also displayed that the ligand-binding site was found in the largest pocket in all 10 proteins tested. Similar was the result yielded from POCKET [58].

Each amino acid (residue) has a distinct impact on the structure and function of a protein. Even if the measured distance between two residues in a protein sequence is long, the spatial distance between them may be short due to protein folding [59–61]. As a result, residues in the sequence that are far from the target residue sequentially, but spatially close, can also have a significant effect on the position of the binding residues. AlphaFold [62] can be considered as one of the major breakthroughs that predicts the tertiary structures of most proteins rather accurately integrating 1D, 2D and 3D protein features. Ultimately, there is a need to consider the spatially neighboring residues for the binding site prediction. Furthermore, the secondary and tertiary structure of the protein also impacts binding, often more significantly than the primary structure.

**Figure 6:**
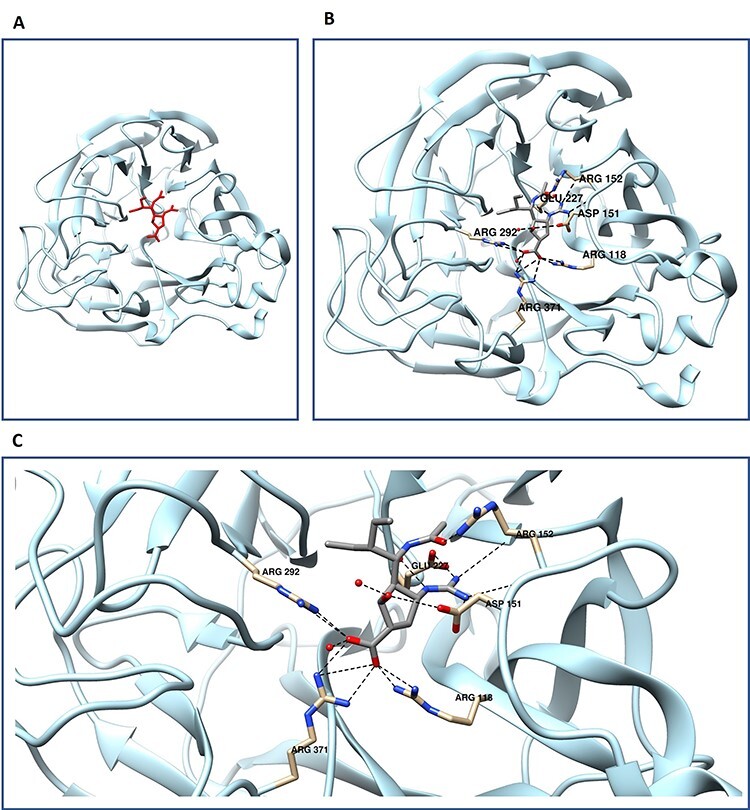
Protein–ligand interactions demonstrated through neuraminidase–peramivir interaction. (**A**) Neuraminidase monomer with peramivir depicted in red. (**B**) view of full monomer with peramivir with hydrogen bonding pairs labeled and displayed in canonical atom coloring. Oxygen colored red, and nitrogen in blue. (**C**) focused view of neuraminidase peramivir hydrogen bonding, PDB code 1L7F.

To further exemplify the concept of ligand-binding site, we take an instance of neuraminidase, an influenza virus protein extensively studied due to its candidacy as a drug target. It is often seen as a good drug target due to the ability to disrupt the life cycle of the virus. Unfortunately, influenza is irritatingly good at manufacturing drug-resistant variations driving the need for constant development of new drugs. One such drug to be developed is peramivir also known as BCX-1812 (sketch shown in Figure 1A. Structurally, peramivir inhibits neuraminidase (interaction shown in Figure 6A) by forming numerous electrostatic interactions including salt bridges and H-bonding [63]. When observing the protein monomer, 11 hydrogen bonds can be found holding peramivir in place greatly increasing the binding affinity for the compound through its enthalpic effects on the system (Figure 6B and C) [6, 54–58]. Although the experimental determination provides the most accurate assignment of the binding locations, it is a time- and labor-intensive process. Computational methods for the detection and characterization of functional sites on proteins have grown in popularity, and as a result, numerous methods have been developed in recent decades attempting to address this issue.

### Binding site prediction methods

Many different approaches to predicting the binding site have been established over the last two decades, based on (i) templates, (ii) energy functions, (iii) geometric considerations and (iv) ML.

The template-based methods attempt to predict the position of binding sites on an input protein using known protein templates. They are based on the assumption that proteins sharing a similar structure can also share a similar function [64]. In comparison to the geometry and energy-based methods, these methods are generally more accurate if a good template can be found [34].

Energy-based approaches rely on the principle to find energetically favorable regions on the protein surface that contain ligand-binding pockets. In most cases, the protein is enclosed in a grid structure, and the interaction energy at each grid point is calculated using one or more probes. The resulting interaction points are then clustered to predict the location of pockets.

In geometry-based methods, the geometry of the molecular surface is analyzed to find surface cavities on the target protein. Based on the algorithm used for cavity localization, Macari *et al*. [34] have divided it into three subcategories: (i) grid system scanning, (ii) probe sphere filling (iii) alpha shape. In the grid system scanning approach, the protein is enclosed into a three-dimensional grid, and latter, if certain geometric conditions are met, are considered as points belonging to a pocket. The accuracy of this method is dependent on the resolution of the grid. Probe sphere–based approaches are based on directly filling pockets or cavities with specific probe spheres. Furthermore, alpha shape methods rely on the computation of Delaunay triangulation [65] to locate voids on the protein surface. The final step of all these methods involves clustering and a ranking procedure of the pockets identified.

The earlier pioneering binding prediction methods use preexisting templates, employing genetic identity, or molecular geometry to predict the binding pockets. COACH and later COACH-D took advantage of the PDB by using previously solved structures as a template for the predictions of the target complex [66, 67]. Another popular server, LigASite and its upgrade LIGSITE^csc^ utilizes geometry by scanning the model on a 3D grid map for convolutions by defining grid points to determine protein solvent locations ultimately identifying potential binding locations [68]. Using these concepts of course has its limitations. Accuracy of the predictions begins to decay as the pairwise identity of the target in respect to the template decreases and often become unreliable as the identity approaches the ‘twilight zone’ [69, 70]. The methods based on templates are rather limiting when encountering novel protein structures. As the field matured, FindSite was developed that also took advantage of structural similarity to find templates. Biologically, the rationale behind using structural and genetically similar proteins as templates for the prediction is due to the idea that as proteins accumulating mutations from years of evolution will result in functional regions having a higher degree of conservation within the same protein family due to risking loss of function [67, 70, 71]. However, cautions must be used when using this method as this conservation is not perfect and there can be a high degree of variability among the residues within the family or the function of highly conserved regions of the protein [70].

But how do these non-ML prediction methods compare to ML? Template-based methods perform well if templates having known binding site information can be found, but do not work if there are no good templates. Machine learning methods can learn from existing data and generalize to new data that are not similar to the training data. Energy function–based approaches are calibrated from a small set of known protein–ligand structures using the function designed by human experts, which may not fit a large amount of data best and cannot generalize well to new data. Similarly, geometry-based methods locate surface cavities on the target protein by analyzing the geometry of the molecular surface, whose precision is largely dependent on the resolution of grid [34]. Another limitation to geometry-based method is the sensitiveness to the scanning direction and to the orientation of protein in the grid system [72]. But machine learning, particularly deep learning methods, can directly learn a parameterized function from a large amount of data integrating multiple sources of information, leading to better accuracy of predicting protein–ligand interaction.

The growing availability of high-resolution protein structures in various databases has opened up new possibilities for machine learning (ML) applications. The basic workflow of existing ML methods to predict binding sites can be divided into five main steps: data acquisition and preprocessing, feature engineering, model development, training–testing, hyperparameter tuning and evaluation. At first, several sources of known protein–ligand binding data are aggregated, and several significant features are extracted to represent the protein and ligand, which are then normalized. Then, ML models are designed to use the input features to predict binding sites, including shallow supervised learning algorithms, artificial neural network, convolutional neural network and ensemble methods (different approaches are further described in detail in Sections `Classical ML methods for binding site prediction' and `Deep learning methods for binding site prediction'). A typical ML workflow is illustrated in Figure 3. We group ML methods into two categories: classical ML methods (non–deep learning methods) and modern deep learning methods to be described separately below.

### Classical ML methods for binding site prediction

Table 1 contains a summary of a list of the classical methods, their machine learning techniques, input features and training/test data, which are reviewed below.

**Table 1:** Classic ML methods to predict protein–ligand binding sites

SN	Approach	Techniques	Features	Database used	Year
1	Oriented Shell Model [73]	Support vector machine	Developed oriented shell model, utilizing distance and angular position distribution	Self-curated	2005
2	SitePredict [74]	Random forest	Predicted small ligand-binding sites mobilizing backbone structure	Self-curated	2008
3	LIBRUS [75]	Support vector machine	Combined ML and homology information for sequence-based ligand-binding residue prediction	Self-curated + FINDSITE’s database	2009
4	Qiu and Wang’s method [76]	Random forest	Used eight structural properties to train random forest classifiers, latter combined to predict binding residues	Q-SiteFinder’s dataset	2011
5	Wong *et al*.’s method [77]	Support vector machine + differential evolution	Classified the grid points with the location most likely to contain bound ligands	LigASite	2012
6	DoGSiteScorer [78]	Support vector machine	Web server for binding site prediction, analysis and druggability assessment	Self-curated	2012
7	Wong *et al*.’s method [79]	Support vector machine	Used SVM to cluster most probable ligand-binding pockets using protein properties	LigASite + self-curated	2013
8	TargetS [80]	Support vector machine + modified AdaBoost	Designed template-free predictor with classifier ensemble and spatial clustering	BioLip	2013
9	Wang *et al*.’s method [81]	Support vector machine + statistical depth function	SVM model integrating sequence and structural information	PDBbind	2013
10	LigandRFs [82]	Random forest	Applied random forest ensemble to identify ligand-binding residues from sequence information alone	CASP9 targets + CASP8 targets	2014
11	Suresh *et al*.’s method [83]	Naive Bayes classifier	Trained Naive Bayes classifier using only sequence-based information	Self-curated	2015
12	OSML [84]	Support vector machine	Proposed dynamic learning framework for constructing query-driven prediction models	BioLip + CASP9 targets	2015
13	PRANK [7]	Random forests	Developed mechanism to prioritize the predicted putative pockets	Astex Diverse set + self-curated	2015
14	UTProt Galaxy [85]	Support vector machine + neural network + random forest	Developed pipeline for protein–ligand binding site predictive tools using multiomics big data	Self-curated	2015
15	Chen *et al*.’s method [86]	Random forest	Proposed dynamic ensemble approach to identify protein–ligand binding residues by using sequence information	ccPDB + CASP9 targets + CASP8 targets	2016
16	Chen *et al*.’s method [87]	Random forest	Predicted allosteric and functional sites on proteins	PDBbind + allosteric DB + CATH DB	2016
17	TargetCom [88]	Support vector machine + modified AdaBoost algorithm	Designed ligand-specific methods to predict the binding sites of protein–ligand interactions by an ensemble classifier	BioLip	2016
18	P2Rank 2.1 [89]	Bayesian optimization	Improved version of P2Rank	Self-curated	2017
19	P2Rank [90]	Random forest	Built stand-alone template-free tool for prediction of ligand-binding sites	Self-curated	2018
20	PrankWeb [91]	Random forest	Online resource providing an interface to P2Rank	Self-curated	2019

In 2013, Wong *et al*. [77] proposed a method for predicting protein–ligand binding sites using support vector machines (SVM). SVM was used to cluster the pockets that are most likely to bind ligands based on geometric characteristics (grid values calculated by LIGSITE and SURFNET that can represent binding site), interaction potential (calculated using the PocketFinder method), offset from protein, conservation score (obtained from a residue-level analysis) and properties surrounding the pockets. The dataset (LigASite) used to train the method faces the same issue as most bioinformatics dataset: imbalance, i.e. the number of positive examples (the grid points of binding site) is much less than the negative examples (the other grid points). To mitigate this problem, undersampling of negative examples was used, which resulted in better performance. Likewise, Integrating Data Selection and Extreme Learning Machine for Imbalanced Data (IDELM) [92] can be implemented to overcome the data imbalance problem. IDELM, which was designed by modifying Extreme Learning Machine (ELM) [93], was reported to have a faster learning capacity in comparison to ELM.

In the same year, Yu *et al*. proposed TargetS [80], a template-free LBS predictor with classifier ensemble and spatial clustering to address the challenge, especially when the target proteins’ 3D structures are unavailable, or no homology models are available in the library. To create discriminative features, protein evolutionary details, predicted protein secondary structure (as determined by PSIPRED [94]) and ligand-specific binding propensities of residues were combined. To address the severe imbalance problem between positive (binding) and negative (nonbinding) samples, an improved AdaBoost classifier ensemble scheme based on random under sampling was used.

Another approach was proposed by Wang *et al*. [81] introducing the statistical depth function to identify negative samples for predicting binding site using sequence and structural information with SVM. In this study, the statistical depth functions were used to determine the depth of the residues and analyze the protein structure. They chose the half-space depth function to calculate the depth of the residues out of a variety of statistical depth functions because the concept and description of the half-space depth are simple and straightforward. Their research revealed that defining a negative sample in this manner was fair and beneficial to model training.

Inspired by the promising performance of SVM, many other ML approaches have been implemented. In 2005, Guo *et al*. [73] introduced a new statistical descriptor, named Oriented Shell Model, that considers the distance and angular position distribution of several structural and physicochemical features. Similarly, Kauffman and Karypis developed a sequence-based approach, called LIBRUS [75], based on SVM in 2009. And in 2012, Volkamer *et al*. published DoGSiteScorer [78], a web-based tool for predicting binding sites and determining druggability. The same SVM technique was applied by Yu *et al*. in their method: OSML [84].

In Suresh *et al*.’s method [83], they implemented Naive Bayes classifier with amino acid residue in membrane protein sequence. Here, they predicted whether the given input is a ligand-binding residue or not using only sequence-based information. They opted Bayesian classifiers since they are resistant to real-world noise and missing values [37].

LigandRFs [82], a sequence-based method for identifying protein–ligand binding residues with RF, was developed by Chen *et al*. In the process of encoding input features, they proposed a hybrid technique to reduce the effects of different sliding residue windows. They also built several balanced datasets, for each of which an RF-based classifier was trained, addressing the high imbalance between ligand-binding sites and non–ligand-binding sites. They discovered that hydrophilic amino acids are more likely to be ligand-binding sites. Besides LigandRFs, RF algorithm was also implemented by Qiu and Wang’s method [76], Bordner [74], PRANK [7], PrankWeb [91] and UTProt Galaxy [85].

### Deep learning methods for binding site prediction

Deep learning methods have grown in popularity in recent years due to their potential in capturing complicated relationships hidden within the data. Several deep learning methods for binding site prediction are summarized in Table 2.

**Table 2:** List of deep learning methods to predict protein–ligand binding sites

SN	Approach	Techniques involved	Feature	Database used	Year
1	DeepCSeqSite [95]	Deep convolutional neural network	Proposed sequence-based approach for ab initio protein–ligand binding residue prediction.	BioLip	2019
2	DELIA [96]	Hybrid Deep neural network + bidirectional long short-term memory network	Designed hybrid deep neural network is to integrate 1D sequence-based features with 2D structure-based amino acid distance matrices.	BioLip + ATPBind	2020
3	Kalasanty [97]	3D convolutional neural network	Designed model based on U-Net’s architecture.	sc-PDB [98]	2020
4	DeepSurf [99]	Deep convolutional neural network + ResNet	Proposed surface-based deep learning approach for protein–ligand binding residue prediction.	scPDB	2021
5	PUResNet [100]	ResNet	Based on deep ResNet + novel data cleaning process.	scPDB	2021

Cui *et al*. proposed DeepCSeqSite [95] for predicting protein-ligand binding residues, a sequence-based method based on a deep convolutional neural network (CNN). Several convolutional layers were stacked to obtain hierarchical features from input. Binding residues belonging to any selected ligand class were classified as positive samples in the training sets, whereas the remainder were labeled as negative samples. Seven types of features are used for the protein-ligand binding residue prediction: position-specific score matrix, relative solvent accessibility, secondary structure, dihedral angle (predicted by ANGLOR [101]), conservation scores, residue type and position embeddings, which are purely derived from protein sequences.

In 2020, a 3D fully CNN (based on an architecture called U-Net) was published for finding druggable pockets on protein surface [97]. U-Net [102] is a state-of-the-art neural network architecture that was initially invented to deal with the 2D medical images. In this method, the task of pocket detection was reformulated as a 3D image segmentation problem. Both the input and output are represented as 3D grids of the same dimensions.

Xia *et al* published a deep learning–based method called DELIA [96], which is a hybrid deep neural network integrating a CNN with a bidirectional long short-term memory network (BiLSTM) to mobilize 1D sequence feature vectors and 2D distance matrices. DELIA’s hybrid neural network architecture is made up of three main modules: (i) feature extractor, (ii) residual neural network (ResNet) and (iii) BiLSTM. To improve the model, oversampling in minibatch, random undersampling and stacking ensemble strategies were used to resolve the problem of the extreme data imbalance between binding and nonbinding residues.

In 2021, Mylonas’ team [99] proposed a binding site prediction method, DeepSurf, based on deep learning architecture. This work is unique in the sense that it mobilizes surface-based representation (implementation of 3D voxelized grids) along with state-of-the-art deep learning architectures to predict potential druggable sites on proteins. After the input features are determined, those grids are imported to a 3D CNN and the resulted ligandability scores of each surface point obtained thus are clustered to create the binding sites.

Recently in late 2021, Kandel *et al*. published a paper called PUResNet [100], which involves the implementation of Deep ResNet as the backbone of the network in their model for the prediction of protein–ligand binding site. This is unique in the prospect of data cleaning process. Here, 3D protein structure of protein is fed into the model as input and probability of voxel belonging to cavity is given as output. Later, these predictions are saved as mol2 files and visualized using molecular modeling software.

## Prediction of protein–ligand binding affinity

In order to be a lead molecule for drug development, a molecule must be able to bind tightly to a target protein; i.e. it must have a high affinity. The degree of attraction between a receptor (e.g. a protein) and its binding partner (e.g. drug or inhibitor) is measured by binding affinity, which can be expressed by the thermodynamic value of dissociation constant (K_d_) or in the case of inhibitors (K_i_). Table 3 demonstrates a variability of different inhibitors acting upon different proteins. SmCI N23A is a mutant variant of the SmCI inhibitor, demonstrating how small changes to an inhibitor can greatly affect K_i_.

**Table 3:** Binding affinity (K_i_) of the SmCI group of inhibitors on three proteins [161].

Inhibitor ligand (protein)	Porcine pancreatic elastase	Trypsin	Bovine carboxypeptidase A	Human carboxypeptidase A1
SmCI	2.66 × 10^−8^	3.81 × 10^−8^	2.83 × 10^−8^	–
rSmCI	1.70 × 10^−8^	3.66 × 10^−8^	9.55 × 10^−8^	2.54 × 10^−8^
SmCI N23A	1.94 × 10^−9^	4.08 × 10^−10^	4.25 × 10^−8^	1.29 × 10^−8^

Here, – is an indication of no data

Predicting a protein–ligand complex’s binding affinity (such as inhibition constant, dissociation constant and binding energy) is critical for efficient and effective rational drug design. However, experimentally measuring protein–ligand binding affinity is time-consuming and complex, which is one of the major bottlenecks of the drug discovery process.

As discussed earlier, the dissociation constant (K_d_) can be used to explain the affinity between a protein and a ligand. The smaller K_d_, the stronger the binding. In case of enzymes and their inhibitors, the inhibitory constant K_i_ is equivalent to K_d_. Further information about thermodynamic measurements of PLIs has been reviewed by Perozzo *et al*. [103].

In computational medicinal chemistry, calculating ligand-binding affinity is an open challenge. The ability to computationally predict binding affinity of small molecules to specific biological targets is extremely useful in the early stages of drug discovery since it allows a mathematical model to determine PLIs. When opposed to conventional experimental methods or computational scoring approaches, ML methods are significantly faster and less expensive.

In the last couple of years, several databases (as discussed in the `Existing databases for AI-driven protein-ligand interaction models' section) have been maintained. The impressive amount of collected experimental data in these datasets can be used to design different deep learning architectures to develop ML-driven ligand-binding affinity prediction methods.

This section is mainly focused on the application of ML in predicting the ligand-binding affinity, which is still an open challenge in computational drug discovery.

### Classical ML approaches for binding affinity prediction

While modern ML libraries include many supervised ML techniques, the majority of ML applications for the development of scoring functions have concentrated on three main techniques: SVM, RF and linear regression. Table 4 lists the methods that have been studied using classical ML techniques.

**Table 4:** List of classical ML approaches to predict protein–ligand binding affinity

SN	Approach	Technique involved	Feature	Database used	Year
1	Deng *et al*.’s method [104]	Kernel partial least squares	Applied knowledge-based QSAR approach + used genetic algorithm-based feature selection method.	Self-curated	2004
2	Ashtawy *et al*.’s method [105]	KNN + SVM + MLR + MARS + RF + BRT	Explored range of scoring functions employing ML approaches utilizing physicochemical features that characterize protein–ligand complexes.	PDBbind	2011
3	CSCORE [106]	Regression	Developed Cerebellar Model Articulation Controller (CMAC) learning architecture.	PDBbind	2011
4	SFCscoreRF [107]	Random forest	Followed random forest approach to train new regression models.	PDBbind + CSAR	2013
5	B2BScore [108]	Random forest	Predicted binding affinity for protein−ligand complexes based on β contacts and B factor.	PDBBind	2013
6	Li *et al*.’s method [109]	Random forest + multiple linear regression	Analyzed the importance of structural features to binding affinity prediction using the RF variable importance tool.	PDBbind	2014
7	Wang *et al*.’s method [110]	Random forest	Predicted the protein–ligand binding affinity based on protein sequence, binding pocket, ligand structure and intermolecular interaction feature set.	PDBbind	2014
8	Cyscore [111]	Linear regression	Improved protein–ligand binding affinity prediction by using a curvature-dependent surface area model.	PDBbind	2014
9	Pred-binding [112]	Random forest + support vector machine	Applied ML algorithms for binding affinity prediction problem based on a large-scale dataset.	PDSP Ki DB + PubChem + DrugBank + ChemSpider	2016
10	Avila *et al*.’s method [113]	ML methods available in SAnDReS	Applied machine learning box interface of SAnDReS to explore the scoring function virtual space (SFVS).	PDBbind + MOAD + BindingDB	2017
11	Ferreira *et al*.’s method [114]	ML methods available in SAnDReS	Predicted Gibbs free energy of binding (ΔG) based on the crystallographic structure of complexes.	MOAD + BindingDB + PDBBIND	2018
12	Kundu *et al*.’s method [115]	GP + LR + MP + SMOR + Kstar + RF	Incorporated Weka 3.6.8 package to select optimum parameters of the ML algorithms.	PDBBind	2018
13	Boyles *et al*.’s method [116]	Random forest + XGBoost	Used ligand-based features to improve ML scoring functions.	PDBbind + CASF	2019
14	RASPD+ [117]	SVM + LR + KNN + SDN + RF + ERF	Introduced fast prefiltering method for ligand prioritization based on ML models.	PDBbind + DUD-E	2020
15	Amangeldiuly *et al*.’s method [118]	RF + SVR + XGBOOST + KNN	Designed prediction method for binding kinetics based on the ML analysis of protein−ligand structural features.	BindingDB + self-curated	2020
16	Wee and Xia’s method [119]	Ollivier persistent Ricci curvature-based ML	Persistent attributes were used as molecular descriptors and further combined gradient boosting tree.	PDBbind	2021

In 2014, Li *et al*. investigated the significance of structural features in binding affinity prediction and discovered that RF can effectively leverage more structural features and more training samples, resulting in better prediction performance than multiple linear regression [109]. Later, in 2016, Shar *et al*. developed a method called Pred-binding [112], where they compared the performance of RF and SVM, and found that both models (RF and SVM) provide a potent K_i_ predictability while avoiding overfitting. Similarly, Wang *et al*. [110] conducted a comparative analysis of affinity prediction for family-specific protein–ligand complex using RF method. Their method predicted the binding affinity using the features like protein sequence, binding pocket, ligand structure and intermolecular interaction.

Inspired by the Cerebellar Model Articulation Controller (CMAC) learning architecture, a method named CSCORE [106] was designed that used a data-driven scoring function for accurate binding affinity prediction. But it had poor interpretability power. It has been noticed that torsion angles play an important role in docking. Despite its significance, it was not considered in this model. In the study reported in B2BScore [108], authors used RF for binding affinity prediction based on β Contacts and B Factor. The key concept of B2Bscore was focused on two physicochemical properties of PLIs: B factor and β contacts, both of which had not previously been used in affinity prediction. Here, the B factor assesses the mobility and flexibility of dynamic atoms in proteins, which is critical in determining the protein’s activity and functions and β contacts are a small fraction of distance-based contacts [120].

Recently, Holderbach’s team published a protein–ligand binding free energy prediction method called RASPD+ [117], which employs the fast prefiltering approach for ligand prioritization, where RF outperforms others. Similarly, Ashtawy *et al*. [105] investigated a variety of various ML methods in combination with physicochemical features of protein–ligand complexes. Ensemble prediction methods RF and Boosted Regression Trees (BRT) were found to be the most effective in predicting binding affinities of protein–ligand complexes. SFCscoreRF [107] and Boyles *et al*.’s work [116] followed the RF approach to train new regression models as well.

In 2018, Kandu *et al*. [115] used the RF and Gaussian process regression algorithms on protein–ligand binding affinity prediction. As part of the feature extraction process, they determined a total of 127 ligand and protein features. For proteins, they used the whole protein rather than just features of pockets and cavities. This is because calculating the features of the cavity necessitates the details of the cavity, which is time-consuming. Similarly, for ligands, all physicochemical properties available in Pubchem [121], as well as a few structural properties measured using a method called Padel Descriptor [122] were included. The Gaussian process, linear regression, multilayer perceptron, sequential minimal optimization (SMO) regression [123], K-star [124] and RF were used to train 2864 instances with 128 features, and they discovered that the RF model was ideally suited to the protein–ligand binding energy prediction problem.

### Deep learning methods for binding affinity prediction

Table 5 summarizes a list of the deep learning methods for the binding affinity prediction. Back in 2015, to predict binding affinity, Ashtawy and Mahapatra provided novel scoring functions that used a large ensemble of neural networks. [125]. For accurate predictions, the bagging- and boosting-based ensemble of neural networks scoring functions was used. According to their research, the proposed neural network–based scoring functions BsN-Score and BgN-Score obtained the best results. They also found that the neural network–based ensemble models outperformed RF models.

**Table 5:** List of deep learning methods to predict protein–ligand binding affinity

SN	Approach	Technique involved	Feature	Database used	Year
1	BgN-Score and BsN-Score [125]	Ensemble neural networks	Assessed the scoring accuracies of two new ensemble neural network scoring functions based on bagging (BgN-Score) and boosting (BsN-Score).	PDBbind	2015
2	Gomes *et al*.’s method [126]	Atomic convolution layer	Developed 3D spatial convolution operation for learning atomic-level chemical interactions.	PDBBind	2017
3	KDEEP [127]	3D convolutional neural networks	Featurized protein and ligand considering eight pharmacophoric-like properties that are used by a three-dimensional CNN model.	PDBbind	2018
4	DeepDTA [128]	Convolutional neural network	Proposed deep learning–based model that uses only sequence information of both targets and drugs to predict drug target interaction binding affinities.	Kinase dataset + KIBA dataset	2018
5	Pafnucy [129]	Deep neural network	Represented molecular complex with a 4D tensor, processed by three convolutional layers and three dense (fully connected) layers.	PDBbind + CASF + Astex Diverse Set	2018
6	OnionNet [130]	Deep convolutional neural network	Constructed modified deep CNN and defined customized loss function to train multiple-layer intermolecular contact features.	PDBbind + CASF	2019
7	Zhu *et al*.’s method [131]	Neural network	Predicted the binding affinity from a given pose of a 3D protein−ligand complex by pairwise function based on neural network.	PDBbind + CASF-2016	2020
8	DeepAtom [132]	3D convolutional neural network	Extracted binding–related atomic interaction patterns automatically from the voxelized complex structure.	PDBbind + Astex Diverse Set	2020
9	Jones *et al*.’s method [133]	3D CNN + Spatial Graph-CNN	Developed fusion models to benefit from feature representations of two neural network models to improve the binding affinity prediction.	PDBBind	2020
10	AK-score [134]	3D CNN ensemble	Used ensemble of multiple independently trained networks that consist of multiple channels of 3D CNN layers.	PDBbind + CASF	2020
11	graphDelta [135]	Graph-convolutional neural network	Designed graph-convolutional neural networks for predicting binding constants of protein−ligand complexes.	PDBbind + CSAR + CASF	2020
12	DeepDTAF [136]	Deep convolutional neural network	Employed dilated convolution to capture multiscale long-range interactions.	PDBbind	2021
13	LigityScore [137]	Convolutional neural network	Designed rotationally invariant scoring functions.	PDBbind + CASF	2021
14	Seo *et al*.’s method [138]	Deep attention mechanism	Employed deep attention mechanism based on intermolecular interactions.	PDBbind + CSAR	2021
15	DEELIG [139]	Convolutional neural network	CNN was used to learn representations from the features.	Self-curated	2021
16	ResAtom System [140]	ResNet + attention mechanism	Implemented ResNet neural network with added attention mechanism.	PDBbind + CASF	2021

In 2018, Jimenez *et al*. presented KDEEP [127], a protein–ligand affinity predictor based on 3D convolutional neural networks (CNN), which have shown promising results across a wide range of datasets. In this study, both protein and ligand were featurized via a voxelized 24 Å representation of the binding site considering different pharmacophoric-like properties. These descriptors were used by a 3D CNN model, which learns the binding affinity of the complex given enough training examples. Once trained, the network could predict previously unseen instances. Similarly, in 2020, Mohammad Rezaei’s team published a research method called DeepAtom [132], which utilized 3D CNN to extract the atomic interaction patterns from the voxelized complex structure.

On the other hand, Ozturk *et al*. proposed a deep learning–based model, DeepDTA [128], which made use of only drug–target’s sequence information. This study introduces a new deep learning–based model for drug–target affinity prediction that utilizes protein and drug character representations.

In 2019, a new method, OnionNet [130], a multiple-layer intermolecular-contact-based CNN was developed for protein−ligand binding affinity prediction. Its input features were based on rotation-free element pair-specific contacts between ligands and protein atoms. Later in 2020, Zhu *et al.* proposed the binding affinity prediction method by pairwise function based on ANN [131]. Basically, it predicts binding affinity from a given pose of a 3D protein–ligand complex and shows that a simple neural network model based on pairwise interatomic distances performs relatively well for binding affinity prediction.

In addition to these, there have been reports on the works based on the ensemble-based approach. In the study reported in AK-score [134], its model used an ensemble of multiple independently trained networks composed of multiple channels of 3D CNN layers to predict a complex’s binding affinity, which significantly improved prediction quality. The ensemble approach has the advantage of requiring no additional network architecture modifications and being easily applied to most existing models. In 2020, Jones *et al*. proposed improved protein–ligand binding affinity prediction with structure-based deep fusion inference [133]. In this project, they developed a midlevel fusion model together with 3D CNN and spatial graph CNN to predict protein–ligand binding affinity. graphDelta [135] is a related method that uses graph neural networks to predict binding affinity.

Wang *et al*. proposed a predictive model called DeepDTAF [136], where the local and global features were generated using only 1D sequence data. (3D structures of proteins, ligands and their complexes were excluded in input representation). It was a successful method for capturing multiscale interactions for protein–ligand binding affinity prediction that merged dilated convolution with traditional convolution. While in the model, developed by Wang’s team, the ResAtom System [140], they implemented ResNet neural network with added attention mechanism. Similar to ResAtom System, Seo *et al*.’s method [138] also employed attention mechanism to protein–ligand complex binding affinity as attention mechanism was able to capture the ligand-binding sites that contributed to the improvement in the prediction.

The Ahmed group earlier this year in 2021 proposed a deep learning approach called DEELIG [139], which used CNN to extract the spatial relationship information. Docked poses or protein–ligand complexes were not used as input in this research. Another similar CNN application in this domain is LigityScore [137] that includes rotationally invariant scoring functions called LigityScore1D and LigityScore3D.

## Predicting and scoring of protein–ligand binding pose (3D structure)

The ligand active conformation is the 3D structure of a ligand when it is coupled to a protein. Binding mode of the ligand/drug is defined as the orientation of a ligand relative to the target in the bound state. Straightforward to grasp, a binding pose is simply a candidate binding mode.

**Figure 7:**
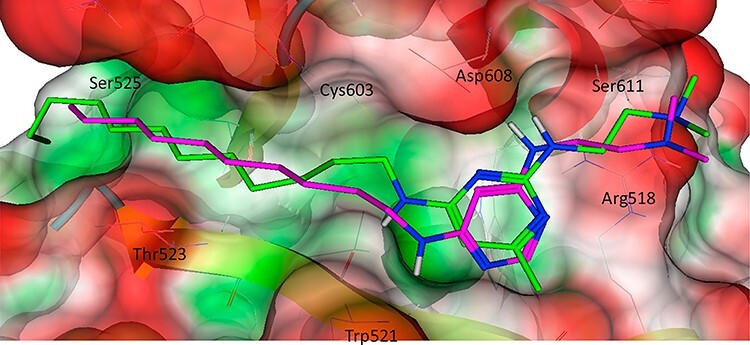
In silico prediction of two similar analogue Inhibitors docked within the binding sight-2 of human dynamin-1 PH domain. (Figure adopted from [141]) Both analogues share very similar intermolecular forces such as H-bonding and yet slight differences in the ligand’s orientation occur.

A visual illustration of protein–ligand binding pose can be found in Figure 7 [141]. It depicts the human large GTPase known as dynamin. Biologically dynamin can excise clathrin-coated vesicles anchored to the membrane for endocytosis of the molecules to their targeted destination within the cell. Due to this, some viruses have learned to use this machinery to gain access to their host cell thus making dynamin a promising druggable target. Figure 7 displays an in silico docking model of two pyrimidine analogues docked within the PH domain of the protein. This demonstrates how ligand pose can vary with extremely minor changes even among structurally similar ligands within the same protein further complicating predictions. In molecular docking, many binding poses are computationally generated and then evaluated using a scoring function. A scoring function is a mathematical model that quantifies the binding stability of the pose, which can be used to rank and select binding poses/conformations. The outcome of a docking run, therefore, is a ligand’s top pose selected according to its predicted binding score. Despite some similarities, it is worth noting that the scoring function here is used to measure the binding stability of a pose, which is conceptually different from the scoring function to quantify the experimental binding affinity such as dissociation constant in `Prediction of protein-ligand binding affinity' section.

The current approaches of predicting protein–ligand binding pose typically have two steps: (i) generating protein–ligand binding poses and (ii) evaluating the poses using a scoring function. As the first step is mostly carried out by some standard/mature docking tools such as AutoDock [142], AutoDock Vina [143], Glide [144], GOLD [145] and Internal Coordinate Mechanics (ICM) [146], the recent development is mostly focused on the second step. Therefore, we mostly review the literature on scoring binding poses in the remaining part of this section below.

The scoring function is one of the most critical aspects of molecular docking. It is used for the selection and ranking of the best poses from the potentially wide list of candidates simulated/generated by the docking algorithms. Despite significant progress, designing a good scoring function capable of reliably predicting binding stability for any conformation remains a challenge in molecular docking.

Generally, scoring functions can be divided into four types: forcefield, empirical, knowledge-based and machine learning–based scoring functions. Please refer to these papers [20, 147–151] for in-depth references to first three kinds. The fourth kind ML-based scoring function is reviewed in the following section.

Here, we mainly focus on classical ML-based scoring functions of scoring protein–ligand docking poses, as well as provide insights into recently introduced deep learning (DL) and reinforcement learning–based scoring methods.

### Classical ML and DL scoring methods for binding pose prediction

With the rapid expansion of ML techniques, ML-based scoring functions have steadily emerged as a promising alternative for scoring docking poses and virtual screening, with majority of them outperforming a wide range of traditional scoring functions. In recent years, the emergence of more powerful deep learning (DL) methods has inspired interest in the exploitation of more precise scoring functions. Table 6 contains a list of the methods described in the subsequent section.

**Table 6:** List of ML methods to predict the binding score of protein–ligand binding pose

SN	Approach	Technique involved	Feature	Database used	Year
1	Ashtawy *et al*.’s method [152]	MLR + MARS + KNN + SVM + RF + BRT	Employed ML approaches utilizing physicochemical and geometrical features characterizing protein–ligand complexes	PDBbind	2015
2	Grudininet *et al*.’s method [153]	Regression	Predicted binding poses and affinities with a statistical parameter estimation	PDBBind + HSP90 dataset + MAP4K dataset	2016
3	Ragoza *et al*.’s method [154]	Convolutional neural network	Trained CNN scoring function to discriminate binding poses using the differentiable atomic grid format as input	PDBbind	2017
4	Ragoza *et al*.’s method [155]	Convolutional neural network	Trained and optimized CNN scoring functions to discriminate between correct and incorrect binding poses	CSAR	2017
5	Nguyen1 *et al*.’s method [156]	Random forest + convolutional neural networks	Used mathematical deep learning for pose and binding affinity prediction	PDBbind	2018
6	Jose *et al*.’s method [157]	Reinforcement learning	An approach to represent the protein–ligand complex using graph CNN that would help utilize both atomic and spatial features to score protein–ligand poses	PDBbind + self-curated	2021

In 2015, to select the optimal pose of a ligand in protein’s binding site, Ashtawy and Mahapatra utilized a variety of machine learning approaches to estimate the difference (root mean square deviation (RMSD)) between a pose and the true structure of a protein–ligand complex [152]. They used protein–ligand complexes’ physicochemical and geometrical features for their prediction and found that ML models trained to predict RMSD values significantly outperform all traditional scoring functions. The best-known empirical scoring function ASP, which is used in the commercial docking software GOLD [145], had a 70% success rate. On the same test set, their top RMSD-based scoring function, MARS::XARG, had a success rate of 80%, indicating a major improvement in docking performance. They also noticed that increase in training set size and number of features increases the performance of scoring functions.

The D3R grand challenges hosted by the Drug Design Data Resource (D3R) has also provided opportunities for computer scientists and bioinformatics researchers to explore the recent advancement in this field. In 2015 D3R Challenge, Grudinin *et al*. evaluated their procedure to score binding poses for protein–ligand complexes using a regression method [153]. They used the affinity and structural data from the PDBBind database to train the model’s free parameters with a regularized regression.

Similarly, in the following D3R Grand Challenge in 2018, Nguyen *et al*. developed a ML-based scoring function [156] to select the poses generated by GOLD [145], GLIDE [144] and Autodock Vina [158]. They created a training dataset of complexes from the PDB after being given a ligand target. Then, using docking software, they re-docked ligands to proteins in those selected complexes. They implemented RF to learn the biomolecular structure and used CNNs to capture topological features. The consensus of the energy values predicted by these two ML strategies was the final predictions for this method.

In 2017, Ragoza *et al*. developed a method using CNN scoring functions to take a detailed 3D representation of a PLI as input automatically learning the main features of PLIs that correlate with binding [155]. Their CNN scoring functions were trained and optimized to distinguish between correct and incorrect binding poses, as well as known binders and nonbinders. The CSAR-NRC HiQ dataset was used as the pose prediction training set, with the addition of the CSAR HiQ Update. They discretized a protein–ligand structure into a grid that is 24 Å on each side and centered around the binding site with a default resolution of 0.5 to handle the 3D structural data as input. Their CNN models were defined and trained using the Caffe deep learning framework [159]. The classes were balanced by sampling the same number of positive and negative examples in each batch after shuffling training data. The CNN model outperformed the Autodock Vina scoring function significantly in terms of intertarget ranking of CSAR poses. They did, however, perform worse in terms of intratarget pose ranking.

Recently, a reinforcement learning–based method for predicting the score of ligand pose has been put forward by the team of Jose [157]. In this method, the agent optimizes the correct pose and can also be trained to locate the binding site. The hypothesis is that training on protein–ligand complexes with known binding poses would aid the reinforcement learning algorithm in approximating the underlying molecular interactions, using the input atomic and spatial features provided as molecule fingerprints. Based on the atomic, spatial and molecular features, this would result in an optimized pose in the desired binding site of the protein of interest. Here, the overall network for reinforcement learning–based protein–ligand docking consists of a GraphCNN layer that represents atomic and molecular properties as a feature vector, as well as an optimization mechanism that approximates the docking scoring function.

## Interconnection of protein–ligand binding site, binding affinity and binding pose

Like all matter, proteins are influenced and shaped by thermodynamic principles that include protein–ligand interactions. Often when characterizing these interactions, intermolecular forces such as hydrogen bonding, van der Waals forces, ion-induced dipoles, desolvation and electrostatic forces are described. All of which directly impact the enthalpy ΔH of the system. Interactions such as H-bonding can net approximately 20 kJ/mol assuming optimal geometry and distance. The intermolecular interactions of the ligand can also dictate the pose of the ligand within the binding pocket. Poses can vary greatly from ligand to ligand due to adjustments of the ligand to bend or twist to accommodate the attractive and repulsive forces involved. It is important to remember molecules are dynamic in nature with varying degrees of flexibility and not static and stiff. Ligands will attempt to orient themselves in the lowest energy conformation possible. Of course, entropy also plays a big role as hydrophobicity of the compounds and the environment in which it is docking can greatly shift what kinds of ligands can bind and how the compound can be accommodated within the binding pocket. The hydrophobicity of the pocket and the ligand can drastically alter the pose of the prospective ligand or outright prevent docking even if the compound is capable of accommodating some of the intermolecular forces needed for specific binding. The entropic penalty of the protein–ligand complex must also not be ignored as it will require most of the free energy that would result from the stabilizing interactions. Because system enthalpy and entropy are so critical in determining the likeliness and orientation of ligand binding, it is commonplace for Gibbs free energy, ΔG to be used as it incorporates both thermodynamic parameters along with accounting for temperature and pressure of the system, which can be valuable when investigating kinetics and molecular dynamics. ΔG is also useful when describing proteins, allowing for equilibrium constants to be derived, in this case, K_d_ or K_i_. The relationship and the effects of the thermodynamic parameters have been reviewed, and it is easy to see how ignoring either thermodynamic principle can greatly impact binding prediction accuracy.

To demonstrate these principles in a more tangible manner, we will use four peptidases with two inhibitors as an example; see Table 3. In this example, we can note that the inhibitor in this case is a family of proteins identified as SmCIs. Biologically, these proteases are regulated through the inhibitory action of its ligand, SmCI proteins. Within Table 3, we see two inhibitors targeting four different peptidases plus one mutant. When comparing SmCI with rSMCI in bovine carboxypeptidase A, we can find an almost 3-fold difference in their K_i_. When looking at trypsin, we find the introduction of somewhat conservative mutation of Asn to Ala causes a drastic magnitudinous change within the K_i_. The substantial change in K_i_ highlights the significance of small thermodynamic changes within the system can have upon ligand binding. This mutation also demonstrates how changes in the system may be counterintuitive if the entire system is not considered.

It is important to be mindful of the system as a whole. Isolating individual parameters such as binding affinity from binding pose will lead to a lackluster prediction, an accurate prediction can only be obtained by accurately representing the controlling natural phenomenon as close as possible. In this case, thermodynamics is the physical link that dictates what and how a ligand will bind to a protein. Therefore, it is essential to integrate the three traditionally separated tasks of predicting binding sites, binding affinity and binding pose together in one comprehensive machine learning system.

## Conclusion and future direction’

In conclusion, based on the findings presented in studies in the above sections, it appears that ML-based PLI prediction methods can reach a higher level of accuracy if we incorporate the use of a large number of physicochemical properties and implement state-of-the-art deep learning techniques.

In the case of binding site prediction, there exists severe data imbalance in the benchmark datasets, making it an imbalanced learning problem, in which the number of samples in different classes (binding or nonbinding) differs significantly. It has been found that applying conventional ML algorithms to imbalanced problems, which presume that samples in different classes are balanced, often results in poor performance. To address this problem, the random undersampling technique can be used to alter the size of the majority class by randomly removing samples from the majority class. Since random undersampling eliminates samples from the original dataset, it provides a sparse training dataset. Moreover, a part of the vital information buried in the removed samples may also be lost simultaneously. Hence, the method of combing multiple random undersampling with classifier ensemble is exploited to balance the sample distribution and at the same time reduce the information loss caused by undersampling. The powerful deep learning approaches have been applied to predict ligand binding sites recently. However, the existing deep learning methods are based on conventional convolutional/recurrent network architectures. Next-generation deep learning architectures based on the attention that has achieved success in protein structure prediction and interpretation [62, 160] shall be developed to further improve the accuracy of LBS prediction.

Protein–ligand binding affinity prediction is still an open challenge in computational drug discovery since it is a highly selective process. It depends on the shape, size, constitutional makeup and physicochemical properties of both drug and its target. Hence, feature selection must be performed with extreme caution, as training a machine is heavily reliant on features. Upon study, it has been found that RF can effectively leverage more structural features and more training samples, resulting in better prediction performance than multiple linear regression. Moreover, RF-based scoring functions are supposed to capture the nonlinear nature of the data more comprehensively than multivariate linear regression (MLR)-based scoring functions. Moreover, among a variety of novel scoring functions using various ML methods in combination with physicochemical features of protein–ligand complexes, ensemble prediction methods RF and Boosted Regression Trees are found to be effective in predicting binding affinities. Regardless of the merits of classical ML algorithms, most of them heavily rely on biological feature engineering to extract explicit fingerprints. Since it is focused on expert information, it is supposed to be biased. Deep learning models, on the other hand, which fall into the descriptor-based category that can automatically extract features from raw data, tend to reduce the bias. It is expected that deep learning methods will play an increasingly significant role in this area.

For achieving a good binding pose, it has been found that picking the right receptor template and reducing the binding pocket size (and hence the size of the search space) as much as possible are critical. Furthermore, research has shown that the flexibility of protein side chains within the binding pocket has no effect in improving the quality of docking poses. Since ML can express nonlinear dependencies between chemical features, it has become an increasingly popular approach for scoring docking poses. RFs, SVMs and neural networks are the algorithms that have been used to solve scoring problems in a number of situations, and they are said to provide more flexibility and expressiveness than traditional empirical scoring methods because they learn both parameters and model structure from data. Furthermore, we see a promising prospect of deep learning and reinforcement learning in the domain of binding pose prediction. It is likely more such methods will be developed in the future. Moreover, as the tasks of predicting binding sites, binding affinity and binding poses are related, advanced deep learning methods of predicting the three simultaneously via multitasking are worth exploring.

**Key Points**

- There is a significant need for improving the prediction of protein–ligand binding site, binding affinity and binding pose to aid drug discovery, drug design and protein function study.
- Artificial intelligence, particularly data-driven machine learning, can significantly advance the prediction of protein–ligand interaction.
- The growing amount of valuable structural and functional data of protein–ligand complexes makes it possible to train highly sophisticated deep learning architectures to predict protein–ligand interactions.
- There is a promising potential of integrating the three traditionally separated tasks of predicting binding site, binding affinity and binding pose together in one comprehensive deep learning system via multitask learning.

## Notes

**Ashwin Dhakal** is a Computer Science graduate student at the University of Missouri-Columbia. His research is focused on analysis and design of machine learning models for predicting protein–ligand interactions.

**Cole McKay** is a Biochemistry graduate student with the University of Missouri-Columbia. His research is focused on structural and biological function of uncharacterized protein domains.

**John J. Tanner** is a professor in the Departments of Biochemistry and Chemistry at the University of Missouri-Columbia. His research interest includes structural biology, X-ray crystallography and enzymes.

**Jianlin Cheng** is a professor at the Department of Electrical Engineering and Computer Science, University of Missouri-Columbia. His research is focused on bioinformatics and machine learning.
